# Mercy Procedure or a Nightmare? Attitude towards Pregnancy Termination among Polish University Students

**DOI:** 10.3390/ijerph19010213

**Published:** 2021-12-25

**Authors:** Maciej Stokłosa, Iga Stokłosa, Mateusz Porwolik, Maciej Bugajski, Gniewko Więckiewicz, Tomasz Męcik-Kronenberg, Magdalena Piegza, Robert Pudlo, Piotr Gorczyca

**Affiliations:** 1 Department and Clinic of Psychiatry, Medical University of Silesia, 42-612 Tarnowskie Gory, Poland; maciej.piotr.stoklosa@gmail.com (M.S.); gniewkowieckiewicz@gmail.com (G.W.); mpiegza@sum.edu.pl (M.P.); rpudlo@sum.edu.pl (R.P.); pgorczyca@sum.edu.pl (P.G.); 2 Department of Ophtalmology, Medical University of Silesia, 40-514 Katowice, Poland; pporwolik@gmail.com; 3 National Research Institute of Oncology, State Research Institute, 31-115 Krakow, Poland; maciek.bugajski@gmail.com; 4 Department of Pathomorphology, Medical Univeristy of Silesia, 41-800 Zabrze, Poland; patolog@interia.pl

**Keywords:** abortion, students, public health

## Abstract

Worldwide, there are about 121 million unintended pregnancies per year. The aim of the study was to assess the attitudes in different groups of students from 40 Polish universities towards pregnancy termination. In total, 9686 university students (20.1% male and 79.9% female) took part in our research. Questions about attitudes towards abortion in all possible circumstances were involved in the survey, which also included a socio-demographic questionnaire. Overall, 94.5% of women and 90.7% of men consider that abortion should be legal in the situation of a threat to the mother’s life, while only 52% of women and 38.7% of men accept it in the case of a hard financial situation for the mother. Most students stand for abortion in the situation of a threat to the mother’s life, in the case of rape, or in the case of a mortal fetus defect. Less than half of the students interviewed consider that abortion should be legal in the case of a hard financial situation for the mother or on the mother’s demand. People who declare a religious affiliation more often disagree with abortion, even in the hardest cases such as a danger to the mother’s life or a mortal fetus defect. The factor that influences the strongest on attitudes towards abortion is being an active Roman Catholic. The majority of students have a pro-choice attitude in respect of pregnancy termination.

## 1. Introduction

By definition, an abortion is the intended termination of a pregnancy by an external intervention, such as a surgical or pharmacological one [1]. It is estimated that this intervention affects about 25% of all pregnancies each year [2]. According to the World Health Organization (WHO), 45% of abortions are performed illegally; 98% of these illegal abortions are conducted in developing countries where socio-economic status is still low. Improperly performed abortions are responsible for 4.7–13.2% of maternal deaths [3]. Moreover, available studies show that modern methods of abortion performed under appropriate conditions by qualified medical personnel are associated with significantly lower mortality than mortality in women during childbirth [4]. Legal restriction of access to abortion in a given country particularly affects women whose financial conditions make it impossible to obtain legal abortion abroad, where termination methods are available, which should be considered as a matter of social justice. Due to abortion restrictions in some countries, women opt for illegally performed abortions that lead to numerous complications, such as infection, hemorrhage, perforation of the uterus, and maternal death [5].

Abortion laws vary from country to country. There are countries where abortion is allowed in all cases, the so-called “at the request” of women (including Australia, Russia, the Czech Republic, Slovakia, Germany, France and Spain), countries where abortion is possible if there are fetal defects, the physical or mental health of the mother is threatened, or the pregnancy is the result of rape (most African and South American countries) and countries where abortion is illegal in all cases (the Vatican, San Marino, Malta) [3]. In Poland, since the fall of communism, for more than 20 years there has been the so-called abortion compromise, according to which an abortion could be performed if the pregnancy resulted from rape, the pregnancy posed a danger to the health and life of the mother, or the fetus had genetic or possibly terminal anatomical abnormalities (e.g., trisomy 21, 18 and 13 pairs of chromosomes, anencephaly). This law was amended in 2020. According to a decision of the Constitutional Court, abortion is possible up to 12 weeks if the life or health of the mother is threatened or it is the result of rape. The provision on the possibility of abortion in the case of fetal defects was found to be unconstitutional [6].

For many years, there has been a debate about whether the right to abortion should be a fundamental right of every woman, or whether the right to life of the unborn fetus should be protected. In most cases, the decision to terminate a pregnancy carries a high psychological stigma, especially for women under the age of 25, as up to 40% of them may develop psychological problems after an abortion (PAD), which may promote the onset of mental disorders, such as post-traumatic stress disorder (PTSD), substance abuse, mood and anxiety disorders, and lead to an increased risk of suicidal behavior [7]. Studies have shown that women who had a pre-pregnancy mental illness are particularly predisposed to mental disorders after abortion. However, this is not true for young women, because in this age group PAD is a consequence of trauma directly related to the abortion experience [7]. A study conducted in Denmark has shown that the birth of a child with a severe birth defect has a statistically significant effect on higher maternal mortality in respect of the period of about 20 years after birth, compared to the birth of a healthy newborn [8]. Studies have shown that keeping an unwanted pregnancy has consequences for the mental health of women later in life. In the population of women, before the legalization of access to abortion, carrying a pregnancy to term was associated with an increased risk of significant depressive episodes compared to women whose pregnancy was planned. These women also obtained a higher score on the Center for Epidemiologic Studies-Depression score (CES-D scale) [9]. It should also be mentioned that women who decided to keep an unwanted pregnancy were more prone to anxiety disorders compared to women who decided to terminate their pregnancy within three years of giving birth [10].

The aim of our study was to determine the attitudes towards abortion among students at Polish universities who are a group of young adults who are of reproductive age, educated, and sexually active. In Poland, according to the statistics of the Central Statistical Office, there were 1,230,254 students, a group which constituted 49.3% of people aged 19–24 [11]. In addition, the aim was to identify the factors that determine the views of the respondents. An important aspect was also to learn about the views of medical students on abortion issues, particularly those who wish to specialize in gynecology, as in many cases these individuals will be dealing with women experiencing difficulties related to pregnancy. Our research raises awareness of views on abortion among people for whom the issue of reproduction is important because of their age and sexual activity. An important, practical aspect of our study is to draw attention to the urgent need for a women’s reproductive health program in Poland and the necessary psychological support for people who have experienced abortion or are in a difficult situation related to a planned delivery.

According to the available literature, this is the first study on attitudes towards abortion conducted on such a large scale among students, i.e., people in education who are of reproductive age and for whom reproductive issues are important or will be in the near future. Furthermore, they are mostly sexually active, so they have to consider the risk of unplanned pregnancies. Moreover, this is a sample of people who will influence the direction in which health promoting policies, reproductive prevention and women’s rights will develop in Poland.

## 2. Materials and Methods

### 2.1. Sample Characteristics

The study involved 9686 university students aged 18–35 years from 40 polish universities who were informed of the purpose of the study and agreed to complete the proprietary abortion questionnaire and socio-demographic questionnaire. We would like to emphasize that the sample size in our study is so far the largest in the field of abortion in Poland.

### 2.2. Distribution of the Survey

After the questionnaire was created, it was reviewed by independent experts in the field of gynecology and obstetrics and public health and given to a control group of 30 people—students from the Medical University of Silesia in Katowice. Finally, in order to ensure the greatest possible anonymity of respondents and to reach as many students as possible, the survey was distributed online via Google forms. The inclusion criterion was university student status. Forty universities were randomly selected from the list of all universities in Poland. Then, the questionnaire was placed in groups on social media associating only students at selected universities with an approval of the group’s administrators. Students had access to the survey for a specified period of time, i.e., October-November 2018. The authors received 9824 completed questionnaires, of which 138 were incorrectly completed, so due to the lack of completeness it was decided to exclude these questionnaires from the study. The Ethical Committee of Medical University of Silesia decided that its approval is not required to conduct research using anonymous, online questionnaires.

### 2.3. The Questionnaire

The questionnaire is provided in Appendix A. The proprietary questionnaire consisted of questions that required the selection of one of the options “for” or “against”, while the socio-demographic section concerned gender, age, field of study and year of study, place of residence and, in the case of medical college students, their planned specialization in gynecology and obstetrics. Respondents were asked their opinion on the hypothetical conditions that would justify abortion—these included abortion in case of the following: (I) threat to the mother’s life; (II) threat to the mother’s health; (III) fatal fetal malformations that may lead to perinatal death or shortly after birth; (IV) non-fatal fetal defects (e.g., Down syndrome); (V) pregnancy resulting from a prohibited act (e.g., rape); (VI) difficult socio-financial situation for the mother; (VII) at the mother’s request if the above conditions do not occur. Respondents had to answer each of the following questions:

- Do you think abortion should be legal in the following cases?
- Would you choose to have this procedure if it involved you or your partner?

In addition, medical school students were asked whether they had chosen or were considering specializing in gynecology and obstetrics, and whether they would have performed an abortion in each of the above cases if they had been gynecologists.

### 2.4. Statistics

The data was analyzed using the STATISTICA 13.3 program (StatSoft, Krakow, Poland). The qualitative variable was compared using chi-square test. For quantitative variables that did not conform to the normal distribution, the Mann–Whitney U-test was used to compare two groups, while for more groups we applied the Kruskal–Wallis test. Spearman rank was used to assess the strength of correlation. The significance level was set at *p* < 0.05.

## 3. Results

The characteristics of the study group are shown in Table 1. There was a trend towards greater female participation in the study—they accounted for almost 80% of the respondents, which is consistent with other studies conducted by the authors using the same methodology. The average age of women participating in the study is significantly higher (*p* < 0.0001; Mann–Whitney U test) than that of men, but it is slightly less than one year. The percentage of declared religiosity is higher among women (Chi2 = 4.84; *p* = 0.027; Chi-square test), but at the significance level of a limit, which casts doubt on the hypothesis in such a large group of respondents and suggests similar religiosity in both groups.

The predominant religion in Poland is Roman Catholicism, which is reflected in the results of this survey—55% of respondents claim to be active Roman Catholics. 6.9% of respondents profess other religions, while 38.1% describe themselves as non-religious. The responses in the above three groups were analyzed as shown in Table 2. Similar trends were observed in the group of non-religious respondents and adherents of religions other than Roman Catholism. Therefore, it was decided to analyze these two groups as one—‘Remainder’—as opposed to the group of religiously active Roman Catholic respondents, hereafter referred to as ‘Roman Catholic’. Proper analysis is provided in Table 3.

Due to the finding of a very strong influence of religiosity on the results of the survey and also due to the unequal number of respondents, it was decided to analyze parameters such as gender or field of study separately for the two main groups (“Roman Catholics” vs. “Remainder”), as shown in Table 4. Nevertheless, similar dependencies were observed in the range of these parameters in both analyzed groups, which will be presented later in the results.

Respondents from the “Roman Catholics” group are significantly more likely to oppose abortion on each of the seven premises. The same tendency is seen in the possibility of abortion when the analyzed premises concern the respondent—the “Remainder” group is much more open to such a possibility. The biggest differences concern the attitude to the references to the difficult socio-financial situation of the mother (VI) and abortion on demand (VII)—there is a threefold difference between the groups on the issue of legality in the above cases and even a fourfold difference when it concerns the respondent. All the differences are statistically significant (*p* < 0.0001; Chi-square test).

“Roman Catholic” men are significantly less in favor of the legality of abortion than women in the two most liberal cases (condition nos. VI and VII). Among religious respondents, men from medical schools are more likely than other groups (including women) to declare themselves willing to perform an abortion when it is a threat to the health and life of the mother (I and II). In both groups analyzed, it is women who more often express the will to legalize abortion in all premises, and slightly more often students from non-medical faculties. In the group that favors legalization of abortion, the age of respondents in all premises is significantly higher than in the other group; the same is true in the group of respondents who allow abortion, if the case involves the respondent or the partner. The above data are presented in Table 5.

The distribution of answers depending on place of residence is shown in Table 4. The lowest (perhaps less frequent) approval of abortion was recorded in villages and the highest (perhaps most frequent) in big cities. Similarly, when the case involved the respondent. All these tendencies in Table 6 are statistically significant (*p* < 0.0001; Chi-square test).

Table 7 and Table 8 shows the responses of students depending on their chosen field of study, divided into “Roman Catholic” and “Remainder” groups.

Table 9 shows the views of students considering specialization in gynecology and obstetrics. Respondents in this group are more likely to accept the legitimacy of abortion in any case under consideration and show a greater willingness to undergo such a procedure than the group not considering this specialization, with the greatest statistical significance of this difference concerning abortion involving fetal defects such as Down syndrome (case IV; *p* = 0.006; chi-square = 7.55).

## 4. Discussion

Religion is the factor that most strongly determines students’ attitudes towards abortion. In our study, it was found that the faithful, especially the followers of the Roman Catholic faith, support the prohibition of abortion in all cases, whether for financial reasons, because of the risk to the health of the mother, because of pregnancy resulting from rape, or because of serious disadvantages of the fetus. Similar results were obtained in a study in Oviedo, Spain, where it was also demonstrated that people belonging to the Roman Catholic Church (which, as in Poland, is the leading group of Christianity in terms of number of adherents) are much less supportive of abortion compared to non-religious people or adherents of other religions [12]. This view has been established for many years, as evidenced by the studies conducted in 1995 and 1985, which were also confirmed in our study [13,14]. It is worth noting that the Roman Catholic religion is the strongest determinant of abortion attitudes compared to other denominations (Judaism, Islam, other branches of Christianity, Buddhism, and others).

Age also correlates significantly with students’ attitudes toward abortion—it has been shown that the older the students are, the more their attitudes toward abortion change toward pro-choice. In a 1998 study that defined attitudes toward abortion as a function of age, race, and religion, it was again found that younger people were more tolerant of abortion. The same study also proved that women have a pro-choice attitude, in contrast to men, who in turn prefer a pro-life option. The results we obtained are similar to those collected 20 years ago by Misra, wherein the female gender is overwhelmingly in favor of the choice “pro-choice” in every case of discussion—fetal defects, risk to the mother’s life, pregnancy resulting from rape, financial reasons, or abortion on demand—compared to the male part of the respondents [15]. Recent multicenter studies conducted in different countries have also found that younger age and female gender are factors that favor pro-choice attitudes [16].

Another demographic factor we examined was the size of the city in which respondents currently live. A statistically significantly greater liberalness regarding abortion was shown by students who lived in larger metropolitan areas compared to clearly smaller towns and villages. A 2002 study in Mexico that looked at young adults and examined their attitudes toward abortion also found that people living in rural areas or the outskirts of large cities were more conservative than those living in larger metropolitan areas [17]. The negative attitude towards abortion in the provinces might be related to the fact that there are more believers in smaller Polish cities, while in more urbanized areas faith plays a smaller role among students [18].

The breakdown of students by field of study also provides insight into trends seen in each industry. Arts students show the greatest tolerance, while respondents studying education, sports science, and medicine prefer the most conservative options. The literature search did not find any studies that would similarly describe attitudes towards abortion depending on the chosen field of study. Most of the studies conducted to date have focused on attitudes towards abortion among specific professional groups, e.g., midwives or health care workers, and individuals directly involved in postpartum care or obstetrics were found to be characterized by favorable attitudes towards abortion [19,20].

In this study, we also distinguished a group of medical students who want to specialize in gynecology and obstetrics. Among them, there was a clear tendency to allow abortion in all cases, not only in cases of fatal fetal defects, but also in cases of unfavorable financial situation of the mother or the so-called abortion on demand. Similar tendencies can be observed in studies conducted among midwives or doctors responsible for pregnancy. This may be due to the fact that they have more knowledge about pregnancy complications due to the specificity of a particular specialization and preparation for their choice, for example, in the case of a fetus with serious birth defects, and they are aware of the dangers to the health and life of the mother, not only in terms of physical health, but also in terms of the psyche of women who are not ready to give birth to a child with a serious defect, or who are not able to cope with reporting a pregnancy after rape. Evidence suggests that women who have chosen to deliver an unwanted pregnancy are at higher risk of developing postpartum depression [21]. In addition, professionals working in obstetrics are aware of the high risk of illegal abortion in uncontrolled conditions, which also contributes to the willingness to help women and perform the procedure in a sterile environment, which is associated with lower mortality and risk of complications [22]. A study conducted in the United States showed that 40% of students who completed an internship in gynecology and obstetrics changed their attitudes toward abortion toward greater availability of legal abortion for women [23].

It is also noteworthy that, in most cases, students support wider access to abortion for all women, while when asked if they would opt for this procedure if their pregnancy, or that of their partner, was related to a specific situation, only a few gave a positive answer. No similar assumptions were found in the available literature, while the results obtained in our study may indicate a general tendency of students to be generally tolerant of abortion, regardless of their own beliefs.

## 5. Limitations

Our study has some limitations. The study used an original questionnaire that had not been previously validated. However, to add value to the study, the questionnaire was previously assessed by independent experts in gynecology and public health. In addition, it should be emphasized that our work is an exploratory research project whose main purpose is to draw attention to the problem and discuss the right to abortion. The population studied was a group of students from Polish universities, hence the general population of young adults who have not completed college education was not taken into account. In our next research projects, we are planning to expand the group of respondents to include people not enrolled in a higher education institution. This will allow us to obtain a representative sample of society and to analyze in more detail the attitudes towards abortion among the group of young adults in the general Polish population.

## 6. Conclusions

In the case of severe or incurable fetal defects, abortion was prohibited. In our study it was proved that among students—that is, people in education, of reproductive age, that is, those who will be interested in starting a family or having children in the near future—there is an overwhelming tendency to consent to abortions in various cases. The vast majority of students, regardless of field of study, age, gender, or religious affiliation, support the possibility of abortion primarily in the case of a threat to the life or health of the mother, a serious or incurable defect in the fetus, or a pregnancy resulting from rape. Less than half of the students support abortion in a difficult social and financial situation for the parents or in the case of abortion on demand. The factors that have the strongest influence on the classification of “pro-life” and “pro-choice” are being Roman Catholic and the gender of the respondents. The respondents expressed their will to have access to methods of abortion in justified cases, which could be a measurable opinion to be taken into account in the legislative process, especially since it is a group of people at reproductive age that could be particularly concerned with this issue in the near future.

## Figures and Tables

**Table 1:** Characterization of the study group. All data contained in table are presented as percentage [%] of answers YES for each question (except for row “Average age”, which is measured in years).

	All	Male	Female	*p* Value
Amount	9689	1947	7739	
%	100	20.1	79.9	
Average age [years]	23.7	23.0	23.9	<0.0001 **
% of Roman Catholic responders	55.1	52.8	55.6	0.027 *
Do you think abortion should be legal in the following cases?				
Threat to mother’s life	93.7	90.7	94.5	<0.0001 *
Threat to mother’s health	79.1	73.3	80.5	<0.0001 *
Fatal fetal defects (perinatal death/shortly after delivery)	88.7	82.9	90.1	<0.0001 *
Fetal defects (e.g., Down syndrome) in which the fetus survives	68.2	56.6	71.2	<0.0001 *
Abortion of pregnancy resulting from a prohibited act (e.g., rape)	82.7	71.1	85.5	<0.0001 *
Abortion in a difficult social and financial situation of the mother	49.3	38.7	52.0	<0.0001 *
Abortion at the mother’s request in the absence of the above	42.9	33.3	45.4	<0.0001 *
Would you decide to undergo this procedure if it concerned you or your partner?				
Threat to mother’s life	86.5	88.2	86.1	0.024 *
Threat to mother’s health	72.0	72.0	72.0	NS
Fatal fetal defects (perinatal death/shortly after delivery)	84.3	78.5	85.7	<0.0001 *
Fetal defects (e.g., Down syndrome) in which the fetus survives	61.1	52.2	63.5	<0.0001 *
Abortion of pregnancy resulting from a prohibited act (e.g., rape)	75.1	64.3	77.9	<0.0001 *
Abortion in a difficult social and financial situation of the mother	37.7	29.8	39.8	<0.0001 *
Abortion at the mother’s request in the absence of the above	33.3	27.8	34.8	<0.0001 *

* *p* value for Chi-square test; ** *p* value for Mann–Whitney U Test

**Table 2:** Attitude to abortion regarding Roman Catholic religion. All data contained in table are presented as percentage [%] of answers YES for each question.

	All	Roman Catholic	Remainder		*p* Value
			Other religions	Non-religious	
Amount	9689	5334	664	3688	
%	100	55.0	6.9	38.1	
Do you think abortion should be legal in the following cases?					
Threat to mother’s life	93.7	89.2	97.3	99.2	<0.0001 *
Threat to mother’s health	79.1	64.4	89.7	96.1	<0.0001 *
Fatal fetal defects (perinatal death/shortly after delivery)	88.7	80.6	95.4	98.5	<0.0001 *
Fetal defects (e.g., Down syndrome) in which the fetus survives	68.2	48.6	83.4	92.5	<0.0001 *
Abortion of pregnancy resulting from a prohibited act (e.g., rape)	82.7	70.5	91.9	97.5	<0.0001 *
Abortion in a difficult social and financial situation of the mother	49.3	26.6	67.1	79.3	<0.0001 *
Abortion at the mother’s request in the absence of the above	42.9	21.6	57.6	72.6	<0.0001 *
Would you decide to undergo this procedure if it concerned you or your partner?					
Threat to mother’s life	86.5	76.0	92.1	98.4	<0.0001 *
Threat to mother’s health	72.0	53.5	83.7	94.3	<0.0001 *
Fatal fetal defects (perinatal death/shortly after delivery)	84.3	72.5	92.2	98.3	<0.0001 *
Fetal defects (e.g., Down syndrome) in which the fetus survives	61.1	39.4	77.0	89.0	<0.0001 *
Abortion of pregnancy resulting from a prohibited act (e.g., rape)	75.1	58.3	85.4	95.6	<0.0001 *
Abortion in a difficult social and financial situation of the mother	37.7	17.8	49.1	67.2	<0.0001 *
Abortion at the mother’s request in the absence of the above	33.3	15.1	42.1	61.8	<0.0001 *

* *p* value for Chi-square test.

**Table 3:** Attitude to abortion regarding religion. All data contained in table are presented as percentage [%] of answers YES for each question.

	All	Roman Catholic	Remainder	*p* Value
Amount	9689	5334	4352	
%	100	55	45	
Do you think abortion should be legal in the following cases?				
Threat to mother’s life	93.7	89.2	98.9	<0.0001 *
Threat to mother’s healt	79.1	64.4	95.1	<0.0001 *
Fatal fetal defects (perinatal death/shortly after delivery)	88.7	80.6	98.0	<0.0001 *
Fetal defects (e.g., Down syndrome) in which the fetus survives	6 8.2	48.6	91.1	<0.0001 *
Abortion of pregnancy resulting from a prohibited act (e.g., rape	82.7	70.5	96.7	<0.0001 *
Abortion in a difficult social and financial situation of the mother	49.3	26.6	77.5	<0.0001 *
Abortion at the mother’s request in the absence of the above	42.9	21.6	70.3	<0.0001 *
Would you decide to undergo this procedure if it concerned you or your partner?				
Threat to mother’s life	86.5	76.0	97.5	<0.0001 *
Threat to mother’s health	72.0	53.5	92.8	<0.0001 *
Fatal fetal defects (perinatal death/shortly after delivery)	84.3	72.5	97.4	<0.0001 *
Fetal defects (e.g., Down syndrome) in which the fetus survives	61.1	39.4	87.3	<0.0001 *
Abortion of pregnancy resulting from a prohibited act (e.g., rape)	75.1	58.3	94.1	<0.0001
Abortion in a difficult social and financial situation of the mother	37.7	17.8	64.4	<0.0001 *
Abortion at the mother’s request in the absence of the above	33.3	15.1	58.7	<0.0001 *

* *p* value for Chi-square test.

**Table 4:** Attitude to abortion regarding religion and gender. All data contained in table are presented as percentage [%] of answers YES for each question.

n = 9686	All	Roman Catholic					Remainder				
%			Male		Female			Male		Female	
		All	Med	Nonmed	Med	Nonmed	All	Med	Nonmed	Med	Nonmed
Amount		5334	183	846	462	3843	4352	163	745	295	3125
Do you think abortion should be legal in the following cases?											
Threat to mother’s life	93.7	89.2	91.3	81.6	94.3	90.1	98.9	98.8	98.0	99.0	99.1
Threat to mother’s health	79.1	64.4	62.3	51.6	62.9	67.5	95.1	93.4	92.9	91.0	96.1
Fatal fetal defects (perinatal death/shortly after delivery)	88.7	80.6	78.9	67.4	82.7	83.2	98.0	96.3	96.5	98.6	98.4
Fetal defects (e.g., Down syndrome) in which the fetus survives	68.2	48.6	31.5	31.3	41.9	54.1	91.1	83.6	84.5	85.9	93.5
Abortion of pregnancy resulting from a prohibited act (e.g., rape)	82.7	70.5	60.5	48.0	68.7	76.0	96.7	91.1	93.5	96.6	97.7
Abortion in a difficult social and financial situation of the mother	49.3	26.6	14.2	17.4	19.0	30.2	77.5	59.3	66.0	66.0	82.0
Abortion at the mother’s request in the absence of the above	42.9	21.6	9.7	13.2	14.5	25.0	70.3	52.8	60.5	50.6	75.3
Would you decide to undergo this procedure if it concerned you or your partner?											
Threat to mother’s life	86.5	76.0	90.5	74.6	77.0	75.4	97.5	97.5	97.7	95.2	97.6
Threat to mother’s health	72.0	53.5	59.3	47.9	48.5	55.0	92.8	94.6	93.2	85.8	93.2
Fatal fetal defects (perinatal death/shortly after delivery)	84.3	72.5	71.2	58.9	68.8	75.8	97.4	96.3	95.3	96.7	98.0
Fetal defects (e.g., Down syndrome) in which the fetus survives	61.1	39.4	22.1	26.0	32.0	44.2	87.3	80.4	82.0	80.8	89.5
Abortion of pregnancy resulting from a prohibited act (e.g., rape)	75.1	58.3	44.2	39.4	47.8	64.6	94.1	89.7	90.1	89.2	95.8
Abortion in a difficult social and financial situation of the mother	37.7	17.8	5.9	13.1	11.5	20.3	64.1	46.7	53.1	49.0	69.7
Abortion at the mother’s request in the absence of the above	33.3	15.1	5.3	11.1	11.01	17.1	58.7	47.0	51.9	41.8	62.9

Med—medical students; Nonmed—other than medical students.

**Table 5:** Attitude to abortion regarding age. All data contained in table are mean age [in years] of answers adequately “YES” or “NO” in each case.

n = 9868	Average Age:		
**Do you think abortion should be legal in the following cases?**	**Yes**	**No**	***p* value**
Threat to mother’s life	23.7	23.3	0.016 **
Threat to mother’s health	23.9	23.1	<0.0001 **
Fatal fetal defects (perinatal death/shortly after delivery)	23.7	23.7	NS
Fetal defects (e.g., Down syndrome) in which the fetus survives	24.1	23.1	<0.0001 **
Abortion of pregnancy resulting from a prohibited act (e.g., rape)	23.8	23.4	<0.0001 **
Abortion in a difficult social and financial situation of the mother	23.2	23.2	<0.0001 **
Abortion at the mother’s request in the absence of the above	24.3	23.2	<0.0001 **
**Would you decide to undergo this procedure if it concerned you or your partner?**	**Yes**	**No**	***p* value**
Threat to mother’s life	23.8	23.5	<0.0001 **
Threat to mother’s health	24.0	23.3	<0.0001 **
Fatal fetal defects (perinatal death/shortly after delivery)	23.8	23.6	0.011 **
Fetal defects (e.g., Down syndrome) in which the fetus survives	24.1	23.1	<0.0001 **
Abortion of pregnancy resulting from a prohibited act (e.g., rape)	23.9	23.3	<0.0001 **
Abortion in a difficult social and financial situation of the mother	24.1	23.3	<0.0001 **
Abortion at the mother’s request in the absence of the above	24.2	23.3	<0.0001 **

** *p* value for Mann–Whitney U Test, NS—non-significant.

**Table 6:** Attitude to abortion regarding place of residence. All data contained in table are presented as percentage [%] of answers YES in each case.

n = 9686	Village	<20 k	20–50 k	50–100 k	100–200 k	200–500 k	>500 k
Do you think abortion should be legal in the following cases?							
Threat to mother’s life	89.9	92.5	94.7	91.9	94.5	95.0	95.8
Threat to mother’s health	68.2	75.5	79.5	74.8	80.9	81.1	85.7
Fatal fetal defects (perinatal death/shortly after delivery)	85.1	87.8	89.9	86.2	89.3	89.7	90.8
Fetal defects (e.g., Down syndrome) in which the fetus survives	53.5	64.1	67.7	65.0	69.4	70.0	77.7
Abortion of pregnancy resulting from a prohibited act (e.g., rape)	76.7	80.5	84.0	80.3	83.9	83.5	86.4
Abortion in a difficult social and financial situation of the mother	28.8	43.9	46.0	43.2	50.4	52.4	63.9
Abortion at the mother’s request in the absence of the above	24.9	37.6	39.6	36.7	45.1	45.2	56.6
Would you decide to undergo this procedure if it concerned you or your partner?							
Threat to mother’s life	79.8	83.5	87.7	84.5	88.0	88.6	89.9
Threat to mother’s health	58.5	68.0	73.0	68.9	71.9	75.1	79.6
Fatal fetal defects (perinatal death/shortly after delivery)	78.6	83.0	85.7	82.1	85.8	85.1	87.2
Fetal defects (e.g., Down syndrome) in which the fetus survives	45.0	56.5	60.0	58.0	61.1	64.2	71.7
Abortion of pregnancy resulting from a prohibited act (e.g., rape)	64.8	72.8	76.3	71.6	75.2	76.7	81.4
Abortion in a difficult social and financial situation of the mother	21.2	32.2	34.1	32.1	39.3	38.6	51.8
Abortion at the mother’s request in the absence of the above	19.1	28.5	30.2	26.9	33.7	34.9	46.2

“k”—thousands of inhabitants.

**Table 7:** Attitude to abortion among Roman Catholics students regarding field of study. All data contained in table are presented as percentage [%] of answers YES for question “Do you think abortion should be legal in the following cases?” in each case.

Roman Catholics	(I) Threat to Mother’s Life	(II) Threat to Mother’s Health	(III) Lethal Fetus Defects	(IV) Fetus Defects	(V) Rape	(VI) Difficult Life Situation	(VII) On Demand
Military	97.1	73.3	91.2	62.5	83.9	41.2	38.2
Social Sciences	95.7	75.8	85.6	61.8	81.7	42.7	35.3
Sport	96.0	73.9	88.5	57.7	80.0	40.9	31.8
Humanistic	88.2	65.6	80.9	52.4	71.8	34.2	29.4
Artistic	88.1	65.1	81.9	43.0	67.0	36.6	27.4
Mining and metallurgy	80.8	52.2	70.4	45.8	59.3	29.6	25.0
Another	89.1	63.4	79.4	54.0	72.0	29.6	24.6
Together-religious	89.2	64.4	80.6	48.6	70.5	26.6	21.6
Medical, non-medical	89.9	66.6	82.4	49.8	73.0	28.1	21.4
Natural	86.8	67.0	83.2	53.6	72.5	30.7	20.8
Economics and management	89.3	65.3	84.3	54.2	75.1	24.6	20.5
Technology	86.2	61.1	76.6	45.3	65.0	23.8	20.4
Agriculture	88.0	70.6	91.7	50.0	79.2	9.1	17.4
Strict sciences—non-technical	85.7	55.3	79.4	39.9	64.0	17.8	14.7
Medical	93.5	62.7	81.6	39.0	66.4	17.6	13.1
Education	86.2	59.8	68.5	32.9	61.6	14.5	11.9

**Table 8:** Attitude to abortion among remainder students regarding field of study. All data contained in table are presented as percentage [%] of answers YES for question “Do you think abortion should be legal in the following cases?” in each case.

Remainder	(I) Threat to Mother’s Life	(II) Threat to Mother’s Health	(III) Lethal Fetus Defects	(IV) Fetus Defects	(V) Rape	(VI) Difficult Life Situation	(VII) On Demand
Artistic	100.0	97.1	99.5	96.4	99.0	92.2	84.1
Social Sciences	99.5	98.5	100.0	95.7	98.6	87.8	82.8
Humanistic	98.7	96.2	98.5	94.2	97.1	84.4	79.3
Natural	99.1	96.2	97.6	92.5	97.6	79.9	72.3
Together-non-religious	98.9	95.1	98.0	91.1	96.7	77.5	70.3
Strict sciences—non-technical	98.2	94.6	96.8	90.7	95.4	78.6	70.0
Technology	98.6	94.0	97.2	89.9	95.7	74.0	69.6
Another	99.5	95.6	98.9	91.1	97.8	78.2	67.3
Economics and management	98.3	93.4	96.5	89.6	96.0	69.0	64.8
Medical, non-medical	98.7	93.9	97.0	88.7	96.0	72.3	64.4
Agriculture	100.0	91.7	100.0	83.3	92.3	75.0	60.0
Education	98.7	94.7	98.7	83.6	98.0	67.9	59.4
Mining and metallurgy	94.7	88.2	90.0	75.0	88.9	68.8	53.3
Medical	98.9	91.9	97.8	85.0	94.6	63.5	51.4
Military	100.0	100.0	100.0	92.3	92.9	53.3	40.0
Sport	100.0	77.8	88.9	77.8	89.0	44.4	37.5

**Table 9:** Attitude to abortion among medical students considering specialization in gynecology and obstetrics. All data contained in table are presented in percentage [%] of answers YES in each case. Differences between two groups in each case was tested using chi-square test.

		Amount (%)	Do You Think Abortion Should Be Allowed on Reference?							As a Gynecologist, Would You Perform an Abortion in Case of Accident?						
Cases:			I	II	III	IV	V	VI	VII	I	II	III	IV	V	VI	VII
Do you consider specializing in gynecology?	NO	1263 (81%)	95.3	75.5	88.1	57.9	78.8	34.9	27.3	84.5	66.9	77.3	49.0	66.5	27.0	22.6
	YES	290 (19%)	95.8	78.0	91.5	67.1	85.3	41.2	34.5	89.2	72.9	84.2	58.4	73.6	35.6	30.1
	*p* value		NS	*	*	*	*	*	*	*	*	*	*	*	*	*

* *p* < 0.01, NS—non-significant.

## Data Availability

Data supporting reported results are available on request from the study team.

## Appendix A. The Questionnaire

Questionnaire.

1. Biological gender: Male/Female
2. Year of birth:
3. Branch of study
  - Agriculture
  - Another
  - Artistic
  - Economics and management
  - Education
  - Humanistic
  - Medical, non-medical
  - Military
  - Mining and metallurgy
  - Natural
  - Social Sciences
  - Sport
  - Strict sciences—non-technical
  - Technology
4. University
5. Year of study
6. Place of residence
  - village
  - town of <20 k inhabitants
  - town of 20–50 k inhabitants
  - town of 50–100 k inhabitants
  - city of 100–200 k inhabitants
  - city of 200–500 k inhabitants
  - city of >500 k inhabitants
7. What is your religion?
8. Are you religiousy active?
  - Yes
  - No
9. Only for medical students: Do you consider specializing in gynecology?
  - Yes
  - No
10. Do you think abortion should be legal in the following cases?
  - Threat to mother’s life: YES/NO
  - Threat to mother’s health: YES/NO
  - Fatal fetal defects (perinatal death/shortly after delivery): YES/NO
  - Fetal defects (e.g., Down syndrome) in which the fetus survives: YES/NO
  - Abortion of pregnancy resulting from a prohibited act (e.g., rape): YES/NO
  - Abortion in a difficult social and financial situation of the mother: YES/NO
  - Abortion at the mother’s request in the absence of the above: YES/NO
11. Would you decide to undergo this procedure if it concerned you or your partner?
  - Threat to mother’s life: YES/NO
  - Threat to mother’s health: YES/NO
  - Fatal fetal defects (perinatal death/shortly after delivery): YES/NO
  - Fetal defects (e.g., Down syndrome) in which the fetus survives: YES/NO
  - Abortion of pregnancy resulting from a prohibited act (e.g., rape): YES/NO
  - Abortion in a difficult social and financial situation of the mother: YES/NO
  - Abortion at the mother’s request in the absence of the above: YES/NO
